# Investigating voice in action teams: a critical review

**DOI:** 10.1007/s10111-020-00646-9

**Published:** 2020-08-05

**Authors:** Hanna L. Krenz, Michael J. Burtscher

**Affiliations:** 1 University of Zurich, Zurich, Switzerland; 2 ZHAW Zurich University of Applied Sciences, Zurich, Switzerland

**Keywords:** Voice, Action teams, Speaking up, Team communication, Recipient of voice

## Abstract

Team communication is considered a key factor for team performance. Importantly, voicing concerns and suggestions regarding work-related topics—also termed speaking up—represents an essential part of team communication. Particularly in action teams in high-reliability organizations such as healthcare, military, or aviation, voice is crucial for error prevention. Although research on voice has become more important recently, there are inconsistencies in the literature. This includes methodological issues, such as how voice should be measured in different team contexts, and conceptual issues, such as uncertainty regarding the role of the voice recipient. We tried to address these issues of voice research in action teams in the current literature review. We identified 26 quantitative empirical studies that measured voice as a distinct construct. Results showed that only two-thirds of the articles provided a definition for voice. Voice was assessed via behavioral observation or via self-report. Behavioral observation includes two main approaches (i.e., event-focused and language-focused) that are methodologically consistent. In contrast, studies using self-reports showed significant methodological inconsistencies regarding measurement instruments (i.e., self-constructed single items versus validated scales). The contents of instruments that assessed voice via self-report varied considerably. The recipient of voice was poorly operationalized (i.e., discrepancy between definitions and measurements). In sum, our findings provide a comprehensive overview of how voice is treated in action teams. There seems to be no common understanding of what constitutes voice in action teams, which is associated with several conceptual as well as methodological issues. This suggests that a stronger consensus is needed to improve validity and comparability of research findings.

## Introduction

Consider a junior employee at a marketing team meeting and a nurse working on an emergency response team who voice their concerns regarding incorrect procedures. Both speak up about work-related topics (Morrison 2014) with the intention of preventing negative consequences for the respective organization (Ashford et al. 2009). However, the consequences in the two work environments differ vastly. In case of the nurse, speaking up about errors and mistakes can help to identify hazards and prevent patient harm, in extreme cases death (Lin and Johnson 2015; Noort et al. 2019). Teams in settings such as this are commonly referred to as action teams that work in high-reliability organizations (e.g., in the high-hazard healthcare, military, or aviation domains; Sundstrom et al. 2000). Here, voice is vital to prevent mistakes that potentially threaten human life and well-being. In contrast, mistakes in other team settings, such as the marketing team in our example, might also lead to adverse outcomes, such as losing customers or damaging the organizations’ reputation, but errors in those settings usually have less severe consequences.

These situations—a junior employee and a nurse stating their concerns—are typical examples for the expression of “voice” at the workplace. In general, voice has been defined as “informal and discretionary communication by an employee of ideas, suggestions, concerns, information about problems, or opinions about work-related issues to persons who might be able to take appropriate action, with the intent to bring about improvement or change” (Morrison 2014, p. 174). From early on, voice research has emphasized the importance of voice in the team context (Van Dyne and LePine 1998). In this context, research has linked voice to improved team learning and better identification of problems and hazardous situations (Morrison and Milliken 2000; Burris 2012; Liang and Farh 2018). As a result, voice is nowadays regarded as an essential part of team communication that is beneficial for organizations (Howell et al. 2015). Particularly, in action teams that work in high-reliability environments, voice is important to maintain safety and team performance (Edmondson 2003; Kolbe et al. 2012). For example, if nurses observe that a surgeon is about to make a mistake such as incising the wrong leg (Kohn et al. 2000), it would be crucial that they express their concerns and thus prevent wrong procedures and patient harm.

Despite the clear importance of voice in action teams, the literature on this topic is rather heterogeneous and characterized by a plethora of different conceptual and methodological approaches to assess voice (Noort et al. 2019). Specifically, we see three potential inconsistencies in the voice literature: (1) the definition of voice, (2) the measurement of voice, and (3) the role of the voice recipient. First, “voice” is usually used interchangeably for “upward voice” or “speaking up”. Moreover, terms like “employee voice”, “upward communication”, or “upward feedback” (Bashshur and Oc 2015; Liang et al. 2012; Milliken et al. 2003) refer to the same or at least to very similar phenomenon as voice.^1^ This is potentially problematic, as researchers seem to use not only different terms, but also different definitions (Morrison 2011).

Second, this apparent heterogeneity with regard to the definition of voice can lead to methodological issues when it comes to investigating voice empirically. Moreover, if the conceptual and methodological treatment of voice systematically differs across studies, aggregation of findings regarding voice in practice would be questionable, as measured constructs are difficult to compare (Burt 1976; Howell et al. 2007). This in turn might hinder the taking of appropriate steps, for instance the development of interventions that can improve effective communication (i.e., including voice) in action teams.

Third, another apparent neglect of voice research concerns the recipient. Simply voicing a concern does not always seem to be enough (Burris et al. 2013; Krenz et al. 2019). To stay with the example of leg surgery, expressing concerns about the surgeon’s procedure does not necessarily prevent patient harm. To actually prevent a serious mistake, the surgeon must react adequately (Morrison 2014). Therefore, highlighting the recipient of voice is important, because the recipient is co-responsible for the voice message’s outcome.

Taken together, we submit that the conceptual and methodological issues of empirical voice research make it difficult to compare and integrate findings across different fields, such as human resource management, industrial relations, and organizational behavior (Wilkinson et al. 2019). This is particularly problematic in action teams, where voice can help to avoid disastrous outcomes including plane crashes or patient deaths (Ashford et al. 2009).

Thus, following the call for bridging the gap between theory and practice (Salas et al. 2018), we aim to review the conceptual (i.e., definitions and role of the recipient) and methodological (i.e., type of measurements and instruments that were used) approaches in previous research on voice in action teams. Specifically, in the present review, we aim to provide an overview of how voice has been treated conceptually and methodologically in studies in the action team literature. We focus on the following questions: (a) How was voice defined? (b) How was voice assessed? and (c) How was the recipient operationalized (i.e., definitions and measurements)?

The goal of the current review of the literature is to provide an overview of how voice has been treated in empirical research, and in doing so, to illustrate potential methodological and conceptual inconsistencies. By identifying these gaps, we aim to contribute to the improvement of future research on voice in action teams as well as to the improvement of future training and interventions based on this research.

### Theoretical background

#### Action team work

Action teams are characterized as teams that are typically composed of members with highly specialized skills (Sundstrom et al. 2000) that perform demanding tasks under high time pressure (Klein et al. 2006). In general, action team work is difficult to predict: situations like heart attacks or fires are time sensitive and cannot be scheduled or determined by the clock (i.e., *epochality* of the action team’s work; Ishak and Ballard 2012). Furthermore, team members’ actions are usually irreversible: Wrong treatments of patients are hard to reverse (i.e., *finality* of the work; Ishak and Ballard 2012). Although the high-reliability settings in which action teams operate involve the management of complex technologies, non-technical skills—also termed human factors—play a crucial role (Weick et al. 1999). For example, research has highlighted the importance of team processes and emergent states in these settings (Baker et al. 2006; Burtscher et al. 2018a, b; Salas et al. 2018). Similarly, team work failure and communication errors have been shown as one of the most frequent reasons for adverse event in both aviation and healthcare (Lingard et al. 2004; Kanki 2019; O’Connor et al. 2008; Williams et al. 2010).

#### Action teams and crew resource management

To address this issue, the aviation industry started to develop crew resource management (CRM) trainings focusing on non-technical skills in the 1980s that are now recommended by all large national and international airlines (Flin et al. 2008) and adopted by other action team environments, such as such as merchant navy, offshore oil industry, nuclear power, and medicine (Flin et al. 2002). Besides leadership, decision making, and situational awareness, communication has been identified as a core non-technical skill (European Union Aviation Safety Agency 2019; U.S. Department of the Air Force 2019). One important function of communication in CRM trainings represents the sending and receiving of information (Kanki 2019). Moreover, CRM training teaches action team members to communicate in an assertive manner and to be able insisting on one’s position until being convinced that other opinions are better (Salas and Cannon-Bowers 2001; Kanki 2019; Foushee and Helmreich 1993). In other words, communication is seen as an important non-technical skill in action teams (Omura et al. 2017; Morrison 2014).

#### Voice

Voice is considered as an essential aspect of verbal communication within and between action teams (Edmondson 2003). Morrison (2014) defined employee voice as “informal and discretionary communication by an employee of ideas, suggestions, concerns, information about work-related issues to persons who might be able to take appropriate action, with the intent to bring about improvement or change” (p. 174). Morrison’s definition, which was based on previous empirical research (Detert and Burris 2007; Van Dyne and LePine 1998; Edmondson 2003), strongly influenced the conceptual frameworks of subsequent voice research (Farh and Chen 2018; Guenter et al. 2017; Noort et al. 2019; Weiss et al. 2018). Nevertheless, as mentioned above, the terms used and the definitions of voice vary in the literature. Especially in fields such as human resource management, organizational behavior, and industrial relations, the understanding of voice differs across disciplines (Wilkinson et al. 2019). Despite this, voice as a construct has attracted increasing research attention (Morrison and Milliken 2000; Pinder and Harlos 2001; Van Dyne and LePine 1998). Voice has been described as extra-role communication behavior (Van Dyne et al. 1995; Morrison 2014) and as distinct from related constructs such as issue selling, whistle blowing, or prosocial organizational behavior (Morrison 2011). Voice is considered as an aid to identifying problems, improving organizational effectiveness, facilitating innovation and learning, and discerning hazardous situations (Burris 2012; Liang and Farh 2018; Morrison and Milliken 2000). These organizational key outcomes led to researchers’ growing interest in what drives voice (Ng et al. 2019). As a result, studies have provided important insights into the antecedents of voice on the individual, team, and organizational level (Bashshur and Oc 2015; Knoll et al. 2016; Morrison 2011) that can motivate (e.g., assertiveness, psychological safety, ethical leadership) or inhibit (e.g., powerlessness, climate of fear, hierarchical structure) employee voice (Morrison 2014). In general, voice research has focused on the sender’s perspective: Studies have investigated personal traits (e.g., proactivity; Grant et al. 2011) or other factors that influence the sender’s willingness to express voice (e.g., leader behavior or voice climate within a work group; Chamberlin et al. 2017; Morrison et al. 2011), thereby identifying barriers and enablers of voice. It is only in more recent years that researchers have begun to consider the recipient’s perspective (Burris 2012; Detert and Burris 2007; Fast et al. 2014).

#### Recipient of voice

One of the most common examples of the importance of voice—or team communication in general—is the tragic airplane crash on the island of Tenerife in 1977, which led to the death of 583 passengers and crew members. Sound recordings from the airplane showed that a flight engineer had expressed his concerns to the captain as to whether the runway was clear for their departure. Unfortunately, the engineer’s voice was not assertive enough to keep the captain from starting the takeoff, which could have prevented the disaster. In the literature, this and similar examples of airplane disasters caused by communication breakdowns are well known (Bienefeld and Grote 2012; Edwards et al. 2009; Flin et al. 2002; Green et al. 2017; Salazar et al. 2014). Surprisingly, despite the clear importance of the role of the captain, who did not respond adequately to the engineer’s voice, this case has never been considered as an indicator of the importance of the recipient of voice. Similarly, the voice literature is somewhat limited due to its neglecting to consider the role of the voice recipient, even though recipients are clearly instrumental in determining the consequences of voice.

Although some studies have pointed out that voice is includes a sender (or “voicer”) *and* a recipient (or “receiver”; Bashshur and Oc 2015), underlining that voice is a two-sided process, there is a lack of clarity regarding the recipient. Morrison (2014) described the recipient of upward voice as a supervisor or a person in an organizationally higher status position, implying that the recipient is someone who can respond and act adequately. Interestingly, studies differ on the question of who *can* be the recipient of voice (Gao et al. 2011). Sometimes the recipient is a person on a higher hierarchical level (Nembhard and Edmondson 2006), and sometimes the recipient is a peer of the sender (Noort et al. 2019). Sometimes, the recipient seems to involve both (supervisor and peers), for example a whole team (Li et al. 2017). Additionally, it remains unclear if the recipient is limited to merely one person (Salazar et al. 2014) or if the recipient(s) can be multiple people (Sherf et al. 2018). In other words, conceptualizations of the recipient are inconsistent. Particularly in action teams, this is problematic, as hierarchical structures (i.e., implying a higher status position of the recipient) are suggested to be a significant barrier for team members’ voice (Friedman et al. 2015; Klein et al. 2006; Weiss et al. 2017). For instance, studies have shown that fear of being punished or rejected by someone with a formally higher status (Milliken and Lam 2009; Morrison 2014) is one of the most prevalent barriers to voicing concerns in healthcare (Morrow et al. 2016; Raemer et al. 2016; Weiss et al. 2017). However, if peers could be voice recipients, too, hierarchical barriers would be less relevant. It is therefore important to clearly define the (group of) recipients of voice in action teams. For that reason, we aim to identify the recipient both in terms of the definition of voice as well as in terms of the actual measurement of voice.

#### Measurement of voice

As one of the pioneers of voice research in action teams, Edmondson (2003) assessed the “ease of speaking up” in operating room teams by means of one single interview question. Edmondson’s approach apparently shaped subsequent research, in that voice has been investigated mostly via self-report (Burris 2012; Edmondson 2003; Nembhard and Edmondson 2006). The self-report measures usually investigate the sender’s or recipient’s perspective on perceived past, actual, or hypothetical voice behavior (Liang et al. 2012; Morrison 2011; Nembhard and Edmondson 2006). For hypothetical voice behavior, studies have used scenarios and vignettes to investigate peoples’ willingness to voice or how people think they would behave (Fast et al. 2014; Sayre et al. 2012). In the present review, we want to explore what instruments (i.e., self-constructed vs. validated scales or items) have been used to assess voice in action teams. Our aim is to investigate the comparability of instruments that have been used, including analysis of their contents.

Addressing the lack of investigating “real behavior”, a growing body of research has investigated team communication—especially in action team context—via behavioral observation (Edmondson 2003; Pattni et al. 2019; Waller and Kaplan 2018). For example, in healthcare, coding schemes such as Co-ACT (Kolbe et al. 2013), ANTS (Flin et al. 2010), and OTAS (Healey et al. 2004) have been developed for observing and assessing team communication behavior. Particularly in action teams, team training (e.g., CRM training) is conducted based on simulated emergency scenarios (Kanki 2019; O’Connor et al. 2008). With the help of these scenarios, team skills (i.e., technical skills and soft skills) can be further developed, including team communication (Schick et al. 2015). According to Marks et al.’s (2000) theoretical framework, team communication can be divided into two parts: communication quality and communication frequency. In the present review, we aim to explore what instruments have been used to assess voice in action teams during behavioral observation. Addressing this question, we use Marks et al. (2000) approach to distinguish between assessment of voice quality and voice frequency.

#### The present review

Taken together, voice represents a significant aspect of communication in action teams. Not only the sender, but also the recipient of voice is important, as the recipient needs to react adequately to the voice message. In the present literature review, we describe how voice has been treated conceptually as well as methodologically, thereby providing a critical perspective of previous voice research in action teams. This can aid identification of potential gaps in the common understanding of voice in action teams that could be considered in future research. Furthermore, this review provides a comprehensive overview of measurement instruments that have been used to investigate voice in action teams. This overview can facilitate the planning of new studies and support researchers and practitioners in their choice of suitable instruments. Moreover, we aim to convey a perspective that emphasizes voice as a dynamic process, considering the sender as well as the recipient. Future training and interventions might benefit from integrating both senders and recipients of voice.

## Method

**Fig. 1:**
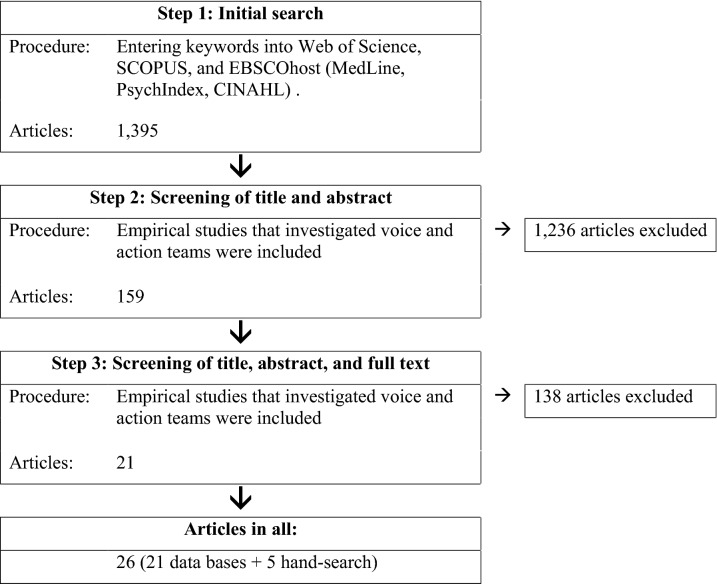
Systematic literature search

We included empirical works published up to May 2019 in our literature review. As our search strategy, we conducted a three-step procedure (Booth et al. 2016) to extract relevant empirical articles that investigated voice and action teams. Figure 1 summarizes the stepwise outcomes of the literature search.

First, to identify relevant empirical studies, we searched the literature using the databases Web of Science Core Collection, SCOPUS, and EBSCOhost (i.e., Medline, PsychIndex, and CINAHL). We combined search terms for voice (i.e., upward voice, lateral voice, speak(ing) up or speak(ing) out, upward communication, upward feedback, employee silence, or organizational silence) with terms for team composition (i.e., team, unit, crew, or department) and terms for action teams that work in high-reliability contexts (i.e., action, medical, emergency, surgical, rescue, fire, military, cockpit, air, or oil). This initial step yielded 1395 articles. In a second step, abstracts and titles were screened by the first author of this review. Each article that did not match the extent of this review was excluded. Concretely, we included empirical, peer-reviewed works that investigated voice (i.e., voice as a distinct measure) quantitatively in an action team context (i.e., professional teams only, no student samples). We excluded the article types meta-analysis, review articles or systematic reviews, conceptual/theoretical articles, qualitative articles, commentary without original results, book chapters without original results, and articles that were written in a language foreign to us (i.e., not English). In cases of ambiguity (e.g., title and abstract did not indicate if voice was examined in the context of an action team), articles were initially included. This second step resulted in 159 articles. In a third step, both authors of this review read titles, abstracts, and full texts carefully to identify relevant articles. Discrepancies were resolved by discussion. We also personally contacted voice researchers and performed a reference check of the existing reviews on voice to identify additional papers. A total of 26 articles met the inclusion criteria, including five articles identified through hand-searching.

## Results

The literature research resulted in 26 studies^2^ published from 2006 to 2019. The studies were conducted in action teams in different domains: aviation (2 studies), healthcare (22), and military (2). The sample included 13 survey studies, 12 simulation studies, and one field study. Sample sizes ranged from 13 (Beament and Mercer 2016) to 1751 (Bienefeld and Grote 2012) participants. Of the 26 studies, 23 studies treated voice as an outcome variable, and three studies treated voice as a predictor, mediator, or moderator variable (Kolbe et al. 2012; Martínez-Córcoles et al. 2018; McClean et al. 2018). Table 1 summarizes characteristics of the studies reviewed. In the following, we use the term ‘voice’ as a collective term for all *variables measured* (e.g., past speaking up behavior, speaking up, challenging authority; for an overview see Table 1).

**Table 1:** Characteristics of the studies included

Authors year	Action team	*n*^a^	Variable measured	Variable	Measurement^b^	Setting	Definition^c^
Bienefeld and Grote (2012)	Aviation	1751	Past speaking up behavior	Outcome	Self-report	Survey	Yes
Bienefeld and Grote (2014)	Aviation	1490	Speaking up	Outcome	Self-report	Survey	Yes
Pian-Smith et al. (2009)	Healthcare	40	Challenging authority	Outcome	Observation	Simulation	No
Kolbe et al. (2012)	Healthcare	62	Speaking up	Predictor	Observation	Simulation	Yes
Sydor et al. (2013)	Healthcare	49	Challenging authority	Outcome	Observation	Simulation	No
Weiss et al. (2014)	Healthcare	54	Speaking up behavior	Outcome	Observation	Simulation	Yes
Robb et al. (2015)	Healthcare	48	Speaking up	Outcome	Observation	Simulation	No
Friedman et al. (2015)	Healthcare	34	Challenging authority	Outcome	Observation	Simulation	No
Raemer et al. (2016)	Healthcare	337	Speaking up	Outcome	Observation	Simulation	No
Beament and Mercer (2016)	Healthcare	13	Verbal challenges	Outcome	Observation	Simulation	Yes
Pattni et al. (2017)	Healthcare	29	Challenging authority	Outcome	Observation	Simulation	No
Weiss et al. (2017)	Healthcare	40	Voice behavior	Outcome	Observation	Simulation	Yes
Weiss et al. (2018)	Healthcare	126	Team member voice behavior	Outcome	Observation	Simulation	Yes
Oner et al. (2018)	Healthcare	70	Speaking up	Outcome	Observation	Simulation	No
Farh and Chen (2018)	Healthcare	402	Member voice	Outcome	Observation	Field	Yes
Kobayashi et al. 2006	Healthcare	240	Willingness to question or challenge authority^d^	Outcome	Self-report	Survey	No
Belyanski et al. (2011)	Healthcare	72	Resident-attending intraoperative communication^d^	Outcome	Self-report	Survey	No
O’Connor et al. (2013)	Healthcare	100	Speaking up about stress, speaking up to seniors	Outcome	Self-report	Survey	Yes
Schwappach and Gehring (2014)	Healthcare	1013	Likelihood of speaking up	Outcome	Self-report	Survey	No
Martinez et al. (2015)	Healthcare	837	Speaking up about patient safety concerns Speaking up about unprofessional behavior	Outcome	Self-report	Survey	Yes
Martinez et al. (2017)	Healthcare	837	Speaking up about patient safety concerns Speaking up about unprofessional behavior Likelihood of speaking up	Outcome	Self-report	Survey	Yes
Schwappach and Richard (2018)	Healthcare	979	Frequency of speaking up-related behaviors Speaking up-related climate	Outcome	Self-report	Survey	Yes
Islam et al. (2019)	Healthcare	564	Employee voice	Outcome	Self-report	Survey	Yes
Alingh et al. (2019)	Healthcare	980	Individual speaking up attitudes	Outcome	Self-report	Survey	Yes
McClean et al. (2018)	Military	174	Promotive and prohibitive voice	Predictor	Self-report	Survey	Yes
Martínez-Córcoles et al. (2018)	Military	161	Critical upward communication	Mo/Me^e^	Self-report	Survey	Yes

^a^Number of participants; ^b^type of measurement; ^c^voice definition provided; ^d^variable name unclear; ^e^moderator/mediator

### Voice definitions

Sixteen of 26 articles (62*%*) provided a definition of voice (i.e., see Table 1), whereas 10 articles (38*%*) did not explicitly clarify their main variable. For example, in the healthcare domain, voice was defined as “stating concerns (e.g., filing a report, sharing concerns with a supervisor or speaking directly with the individual(s) involved) rather than saying nothing” (Martinez et al. 2015, p. 1). In contrast, one study that was set in the aviation domain provided the following definition: “Speaking up in high-risk contexts is defined as an upward voice directed from lower to higher status individuals within and across teams, that challenges the status quo, to avert or mitigate errors” (Bienefeld and Grote 2012, p. 1). Appendix I provides an overview of all voice definitions.

Definitions included aspects such as sender, recipient, how voice should be made, context, content, and intention of voice. Of these aspects, only voice content was commonly mentioned in every definition. The proposed content of voice messages ranged from very precise (“individual’s willingness to inform attendings when they are struggling or stressed or when they have made an error”; O’Connor et al. 2013, p. 426) to less precise (such as the voice content proposed by Morrison (2011) describing “suggestions, ideas, opinions or concerns”; Weiss et al. 2018, p. 390). Notably, voice content was often associated with a critical nature that challenges actions and opinions of persons in higher status positions (Weiss et al. 2018) or, more generally, challenges the status quo (Bienefeld and Grote 2012).

### Measurements

In our review, we distinguished between two types of measurement: self-report measures (*n *= 13) and behavioral observation (*n *= 13). Self-report was based mainly on self-assessments, whereas behavioral observation referred to an external evaluation (i.e., no self-assessment).

#### Self-report

Overall, self-report studies assessed 18 voice variables by means of 25 instruments (for an overview, see Table 2). Instruments that were used to investigate voice in action teams varied from validated scales and items (*n *= 11) to self-constructed scales and items (*n *= 7). Some scales were slightly adapted or modified versions of already validated scales (*n *= 3), whereas other scales represented a mixture of self-constructed items and validated items from already existing scales (*n *= 4). Three of 25 measurement instruments contained one single item to assess voice. The number of items on the instruments ranged from 1 to 27 (*M *= 7.27; SD= 7.34).

**Table 2:** Overview of self-report measures

Author, year	Variable measured	Scale/instrument	Vignette	Item number	SC^a^/MV^b^/V^c^	Source of scale/instrument	Key topic
Bienefeld and Grote (2012)	Past speaking up behavior	Item	–	1	SC + V	Bienefeld and Grote (2012) (i.e., pilot-tested)	Past
Bienefeld and Grote (2014)	Speaking up	Item (i.e., safety critical scenario)	Yes	1	SC + V	Bienefeld and Grote (2014) (i.e., pilot-tested)	Likelihood/willingness
Kobayashi et al. (2006)	Questioning/challenging authority	Attitudes toward communication and patient safety	–	22	MV	Modified Operating Team Resource Management Survey (Helmreich and Merritt)	Attitudes
		Effect of personal values on one’s decision to challenge one’s superior	Yes	16	SC		Attitudes
		Item (i.e., “threshold” for challenging scenario)	Yes	1	SC		Likelihood/willingness
Belyanski et al. (2011)	Resident-attending intraoperative communication	Attitudes toward speaking up and hierarchical barriers (residents/attendings)	–	27/26	SC		Attitudes
O’Connor et al. (2013) ^d^	Speaking up	Speaking up about stress and speaking up to seniors	–	12	V	O’Connor et al. (2012); O’Connor and Long (2011)	Attitudes
Schwappach and Gehring (2014)	Likelihood of speaking up	Items	Yes	4	SC		Likelihood/willingness
Martinez et al. (2015)	Speaking up about patient safety	Speaking Up Climate for Patient Safety (SUC-Safe)	–	6	SC + V	Some items adapted by the SAQ (Sexton et al. 2006)	Climate
	Speaking up about unprofessional behavior	Speaking Up Climate for Professionalism (SUC-Prof)	–	7	SC + V	Guided by the Medical Student Safety Attitudes and Professionalism Survey (Liao et al. (2014))	Climate
Martinez et al. (2017)	Speaking up about patient safety concerns	SUC-Safe	–	6	V	Martinez et al. (2015)	Climate
	Speaking up about unprofessional behavior	SUC-Prof	–	7	V	Martinez et al. (2015)	Climate
	Likelihood of speaking up	Items (i.e., traditional safety threat)	Yes	5	SC		Likelihood/willingness
		Items (i.e., professionalism-related safety threat)	Yes	8	SC		Likelihood/willingness
Schwappach and Richard (2018)	Frequency of speaking up-related behaviors	Perceived concerns	–	3	V	SUPS-Q, Richard et al. (2017)	Past
		Withholding voice	–	4	V	SUPS-Q, Richard et al. (2017)	Past
		Speaking up	–	4	V	SUPS-Q, Richard et al. (2017)	Past
	Speaking up-related climate	Psychological safety for speaking up	–	5	V	SUPS-Q, Richard et al. (2017)	Climate
		Encouraging environment for speaking up	–	3	V	SUPS-Q, Richard et al. (2017)	Climate
		Resignation toward speaking up	–	3	V	SUPS-Q, Richard et al. (2017)	Climate
Islam et al. (2019)	Employee voice	Employee voice	–	6	V	Van Dyne and LePine (1998)	Current
Alingh et al. (2019)	Individual speaking up attitudes	Communication openness scale	–	3	V	Smits et al. (2008)	Attitudes
McClean et al. (2018)	Promotive voice	Promotive voice	–	3	MV	Liang et al. (2012)	Current
	Prohibitive voice	Prohibitive voice	–	3	MV	Liang et al. (2012)	Current
Martínez-Córcoles et al. (2018)	Critical upwards communication	Items	–	3	SC		Current

^a^Self-constructed; ^b^modified version of a validated scale; ^c^validated scale; ^d^article did not provide scale items

By analyzing the instruments used in self-report studies, we identified five key topics: past voice behavior (*n *= 4), attitudes toward voice (*n *= 5), current voice behavior (*n *= 4), voice climate (*n *= 7), and likelihood/willingness to voice (*n *= 5). In contrast to the other key topics, investigation of the likelihood/willingness to voice was always related to a vignette that illustrated typical situations (i.e., for the respective action team context) in which the expression of voice would have been necessary (Bienefeld and Grote 2014; Martinez et al. 2017; Schwappach and Gehring 2014). Table 3 shows example items for the five key topics.

**Table 3:** Example items for self-reports illustrating key subjects

Authors, year, page	Item	Key topic
Schwappach and Richard (2018), online supplementary appendix	“Over the last 4 weeks, how often did you bring up specific concerns about patient safety?”	Past
Belyanski et al. (2011), p. 389	“Residents should not challenge or question attending during OR cases”	Attitudes
Martínez-Córcoles et al. (2018), p. 6	“I can express my disagreements with my superior freely”	Current
Martinez et al. (2015), p. 5	“Nurses input is well received in my clinical area”	Climate
Bienefeld and Grote (2014), Appendix	*Description of a boarding scenario in aviation:* “If you were to encounter this situation on one of your flights, what would you do? Please indicate your willingness to speak up to the captain on the scale below from 0 (I will remain silent) to 100 (I will definitely speak up)”	Likelihood/ willingness

#### Behavioral observation

Each of the 13 behavioral observation studies focused on a single voice variable that was assessed by a specific measurement instrument. Moreover, the instruments that were used to assess voice were mainly modified versions of already existing scales (*n *= 9). One study used a validated instrument (Kolbe et al. 2012), two studies used self-constructed instruments (Pian-Smith et al. 2009; Raemer et al. 2016), and one study did not report the instrument (Farh and Chen 2018). For a detailed description of studies that investigated voice by means of behavioral observation, see Table 4.

**Table 4:** Overview of behavioral observation measurements

Author, year	Variable measured	Scale/rating system	Focus	QU^a^/FR^b^	SC^c^/MV^d^/V^e^	Source/based on literature
Pian-Smith et al. (2009)	Challenging authority	Scoring system for language	Language	QU/FR	SC	Pian-Smith et al. (2009)
Kolbe et al. (2012)	Speaking up	Co-ACT	Event	FR	V	Kolbe et al. (2012, 2013)
Sydor et al. (2013)	Challenging authority	Modified version: Scoring system for language	Language	QU	MV	Pian-Smith et al. (2009)
Weiss et al. (2014)	Speaking up behavior	Modified version: Co-ACT that differentiates between suggestion-, problem-, opinion-, and doubt-focused voice	Event	FR	MV	Kolbe et al. (2013); cf. Morrison (2011)
Robb et al. (2015)	Speaking up	Adapted version: influence tactics	Event	QU/FR	MV	Kipnis et al. (1980)
Friedman et al. (2015)	Challenging authority	Modified advocacy-inquiry score (mAIS)	Language	QU	MV	Bould et al. (2015); Pian-Smith et al. (2009)
Raemer et al. (2016)	Speaking up	Incidence of speaking up for three events	Event	FR	SC	
Beament and Mercer (2016)	Verbal challenges	Adapted verbal challenges grading score	Language	QU	MV	Pian-Smith et al. (2009)
Pattni et al. (2017)	Challenging authority	Modified advocacy-inquiry score (mAIS)	Language	QU/FR	MV	Bould et al. (2015); Pian-Smith et al. (2009)
Weiss et al. (2017)	Voice behavior	Modified version of Co-ACT	Event	FR	MV	Kolbe, Burtscher, and Manser (2013)
Weiss et al. (2018)	Team member voice behavior	Modified version of Co-ACT	Event	FR	MV	Kolbe, Burtscher, and Manser (2013)
Oner et al. (2018)	Speaking up	Modified version: Scoring system for language	Language	FR	MV	Pian-Smith et al. (2009)
Farh and Chen (2018)	Member voice	–	Event	FR	n.r.^f^	Morrison (2011), Edmondson (2003, Nembhard and Edmondson (2006)

^a^Quality; ^b^frequency; ^c^self-constructed; ^d^modified version of a validated scale; ^e^validated scale; ^f^not reported

Measures in studies that examined voice via behavioral observation were divided into two main categories: event-focused (*n *= 7) and language-focused (*n *= 6). Event-focused voice refers to the identification of voice itself (i.e., whether or when did voice occur), whereas language-focused refers to studies in which the formulation of voice is analyzed in more detail (i.e., rating of the assertiveness of voice). Except for two studies (Raemer et al. 2016; Robb et al. 2015), the theoretical approach to event-focused voice was mainly based on Morrison (2011, 2014) and involved identifying suggestion-, problem-, doubt-, or opinion-focused expressions of voice (Farh and Chen 2018; Weiss et al. 2014) through behavioral observation. For example, Kolbe et al. (2012) coded speaking up by expressions such as “Are you sure you want to intubate right now?” (p. 3). Event-focused coding was mainly used to predict voice frequency as an outcome (*n *= 6). In only one case was event-focused coding used to also predict voice quality (Robb et al. 2015). Studies using a language-focused approach were all based on Pian-Smith et al.’s (2009) scoring system. This system initially considered challenges towards the: (a) attending surgeon, (b) nurse, or (c) anesthesiologist. Pian-Smith et al. distinguished between five levels of assertiveness, ranging from saying nothing to verbalization of advocacy and inquiry statements (e.g., “I’m wondering about risks of doing this when there’s a low platelet count. How do you decide how to proceed?”; p. 87). This scoring system was usually slightly modified in more recent studies (Friedman et al. 2015; Pattni et al. 2017). In contrast to event-focused voice, language-focused coding was used to predict voice quality as an outcome (*n *= 3) or a combination of voice quality and frequency (*n* = 2). In only one case was language-focused voice used to assess voice frequency (Oner et al. 2018).

### Recipient of voice

In the present review, we distinguished three categories of recipients. The first category comprises recipients who clearly have a higher status (HS) in terms of being in higher positions in the organization. For example, “Junior team members should not question the decision made by *senior personnel*” (Kobayashi et al. 2006, p. 279). The second category considers a recipient in the sender’s “group” (G) who can be either a peer *or* a higher-status person, or also just the whole group (i.e., more detailed description of the recipient is lacking). For example, “In *my clinical area*, it is difficult to speak up if I have a patient safety concern” (Martinez et al. 2015, p. 5)”. The third category was used when the recipient was not mentioned and/or not identifiable (i.e., no specific recipient, or NSR). For example, “Over the last 4 weeks, how often did you address an error which—if uncaptured—could be harmful for patients?” (Schwappach and Richard 2018, Appendix). The three categories were not mutually exclusive, as particularly self-report studies employed scales in which the recipient varied between items. Table 5 shows the operationalization of the voice recipient in the studies. Operationalization of the recipient refers to holding definitions against actual measurements. As three studies (Martinez et al. 2015, 2017; Schwappach and Richard 2018) employed more than one instrument to assess voice, we identified 30 instruments in total (i.e., instead of 26). That is why in four cases, definitions were used twice (i.e., so as to be able to compare the additional instruments).

**Table 5:** Operationalization of voice recipient(s)

Authors	Variable(s) measured	Definition^a^	Measurement^b^	Fit^b^
Bienefeld and Grote, (2012)	Past speaking up behavior	HS	HS	Yes
Bienefeld and Grote (2014)	Speaking up	HS	HS	Yes
Pian-Smith et al. (2009)	Challenging authority	–	HS + G	No
Kolbe et al. (2012)	Speaking up	NSR	G	No
Sydor et al. (2013)	Challenging authority	–	HS	(Yes)
Weiss et al. (2014)	Speaking up behavior	NSR	HS + G	–
Robb et al. (2015)	Speaking up	–	HS	(Yes)
Friedman et al. (2015)	Challenging authority	–	HS	(Yes)
Raemer et al. (2016)	Speaking up	–	HS + G	No
Beament and Mercer (2016)	Verbal challenges	G	HS	No
Pattni et al. (2017)	Challenging authority	–	HS	(Yes)
Weiss et al. (2017)	Voice behavior	NSR	HS	No
Weiss et al. (2018)	Team member voice behavior	HS	HS + G	No
Oner et al. (2018)	Speaking up	–	HS	(Yes)
Farh and Chen (2018)	Member voice	NSR	HS	No
Kobayashi et al. 2006	Willingness to question or challenge authority	–	HS + G+NSR	No
Belyanski et al. (2011)	Resident-attending intraoperative communication	–	HS + G+NSR	No
O’Connor et al. (2013)	Speaking up about stress/to seniors	HS	HS + NSR	No
Schwappach and Gehring (2014)	Likelihood of speaking up	–	HS + G	No
Martinez et al. (2015)	Speaking up about patient safety concerns	HS + NSR	G	No
	Speaking up about unprofessional behavior	HS + NSR	G + NSR	No
Martinez et al. (2017)	Speaking up about patient safety concerns	NSR	G	No
	Speaking up about unprofessional behavior	NSR	G + NSR	No
	Likelihood of speaking up	NSR	HS + G	No
Schwappach and Richard (2018)	Frequency of speaking up-related behaviors	NSR	G + NSR	No
	Speaking up-related climate	NSR	HS + G+NSR	No
Islam et al. (2019)	Employee voice	NSR	G	No
Alingh et al. (2019)	Individual speaking up attitudes	G	HS + NSR	No
McClean et al. (2018)	Promotive and prohibitive voice	NSR	G	No
Martínez-Córcoles et al. (2018)	Critical upward communication	NSR	HS	No

^a^HS = higher status; G = group; NSR = no specific recipient; - = no definition; ^b^x = fit; yes = fit between definition and measurement; no = no fit between definition and measurement; (yes) = without definition and with term indicating hierarchical relationship between sender and recipient

#### Operationalization of the voice recipient

In 20 available definitions of voice, the recipient was explicitly mentioned as a higher-status person (*n *= 4), as someone in the group (*n *= 2), as higher status *and* not specified (*n *= 2), or not specified at all (*n *= 12). Compared to voice definitions, actual measurements of voice involved even more different combinations of voice recipients: higher status (*n *= 11), higher status and group (*n *= 6), higher status, group, and recipient not specified (*n *= 3), higher status and recipient not specified (*n* = 2), group (*n *= 5), and group and recipient not specified (*n *= 3). Finally, in only two of 30 measurements (7*%*), the recipient was both explicitly mentioned in the definition of voice and operationalized accordingly. Considering studies that did not provide a definition of voice but used terms for their measured variables such as “challenging authority” and “speaking up” that indicated hierarchical relationships between sender and recipient, there would be five additional cases in which the recipient was adequately operationalized (see Table 5).

## Discussion

We reviewed original quantitative studies that investigated voice in action teams. In sum, our review of the literature yielded 26 separate studies, which were predominantly conducted in healthcare teams. Our findings show that only 16 out of 26 articles contained an explicit voice definition. An equal number of studies (i.e., 13 each) investigated voice by means of self-report and by behavioral observation. Whereas behavioral observation instruments were consistently based on either of two theoretical approaches, self-report instruments were more heterogeneous. Another issue concerns the voice recipients; recipients were not consistently defined or measured, which resulted in poor operationalization of the voice recipient. This is the first time that the treatment of voice in action teams has been systematically analyzed in terms of conceptual as well as methodological approaches. Our findings indicate several challenges and implications for voice research in action teams, which we discuss in the following sections.

### Definition of voice

More than one-third of the articles reviewed do not provide a definition of voice. This is problematic, as in general variables that are investigated in research articles should be clarified and distinguished from similar constructs in advance (Suddaby 2010). A possible explanation for not defining the term voice could be that at a first glance, “upward voice”, “speaking up”, or “challenging authority” might appear to be self-explanatory and therefore not worth describing in more detail. However, different researchers’ approaches of that are in line with varying definitions and interpretations of voice (Wilkinson et al. 2019) demonstrate that voice as a concept is not self-explanatory. There is a great need for definitions to adequately operationalize the construct of voice and thus obtain valid and comparable measurements (Moosbrugger and Kelava 2012).

In addition, the aspects mentioned in definitions of voice vary considerably. For example, studies mention sender, recipient, how voice should be made, context, and intention of voice in different combinations and use them very inconsistently. Nevertheless, voice content represents the only common aspect of every definition. Although voice content is always mentioned, there is much room for interpretation, in particular when definitions were borrowed from Morrison (2014) (e.g., “suggestions, concerns, or information about a team’s task”; Farh and Chen 2018, p. 97). This “umbrella construct” of voice (Morrison 2011) is problematic, as more nuanced forms of voice content that specify the type of information conveyed would be valuable (Morrison 2011). Notably, one study—McClean et al. (2018)—examined the effects of different types of voice, such as promotive and prohibitive voice. Whereas promotive voice is associated to the expression of suggestions or new ideas, prohibitive voice is associated to the expression of concerns about incidents that can be harmful to the organization (Liang et al. 2012). Although the distinction between promotive and prohibitive voice has become established in the voice literature (see the meta-analysis in Chamberlin et al. 2017), this distinction is mostly absent in action team research. In action teams, however, we see a potential benefit of explicitly distinguishing voicing a concern from voicing an idea, as the former is more likely to prevent harm and might be more urgent (Lin and Johnson 2015).

### Measurements of voice

Types of voice measurements (i.e., self-report measures and behavioral observation) were equally distributed in the studies in our literature review. The investigation of voice by means of self-report appears to be highly inconsistent, as the instruments varied from self-constructed items to validated scales.

In sum, nearly the half of the self-report studies used self-constructed instruments or even single items to investigate voice (Bienefeld and Grote 2012; Martinez et al. 2017). Although these self-constructed voice instruments are usually developed by experts in the respective field (Belyanski et al. 2011), comparability of different voice measurements is lacking, which leads to fragmentation of research findings.

In addition to the high inconsistency of self-report instruments, the contents of the instruments included different key topics, such as past voice behavior, attitudes toward voice, current voice behavior, voice climate, and likelihood/willingness of voice. These key topics make it difficult to integrate the contents of voice instruments across studies. This is problematic, as content differences between instruments lead to the conclusion that single instruments are possibly not relevant and representative enough to measure the same target construct (i.e., voice), suggesting a lack of content validity (Haynes et al. 1995).

Besides the inconsistency of self-report measures regarding construction and content, the use of post hoc measures as reliable data may be problematic when examining team dynamics (Kolbe and Boos 2019; Kozlowski 2015; Noort et al. 2019). For example, self-report data can be influenced by individuals’ espoused theories (theories involving their values, attitudes, and beliefs; Argyris and Schon 1974). These espoused theories do not necessarily represent actual theories-in-use (effectively applied theories; Argyris and Schon 1974). This discrepancy between how people think they would behave and how they actually behave is particularly noticeable when it comes to potentially threatening and embarrassing issues, which are prevalent in many voice situations (Argyris 1980). Thus, if research relied solely on self-report data, there would be still a lack of insight regarding when, whether, and how people show voice (Krenz et al. 2020).

Our findings show that the studies that investigate voice by means of behavioral observation can be divided into two categories: event-focused and language-focused. In most studies, event-focused voice referred to voice frequency as an outcome (Weiss et al. 2014), whereas language-focused voice was related to the assessment of voice quality (i.e., assertiveness; Pian-Smith et al. 2009). Moreover, 12 of 13 studies were conducted in simulated settings, usually representing parts of training or an intervention for professional healthcare teams (Raemer et al. 2016; Weiss et al. 2018). As a notable exception, Farh and Chen (2018) observed the voice behavior of medical teams conducting routine surgeries in the field. Although simulations are created as realistically as possible, team members’ behavior may differ from their behavior during real emergency situations, as they perceive training as psychologically safer than their work with real patients (Edmondson and Lei 2014). Thus, simulations could ease the barrier of speaking up (Weiss et al. 2018). Nevertheless, compared with self-report, the investigation of voice in action teams by means of behavioral observation (i.e., in simulated training courses or field settings) seems to be advantageous, as they capture real-time behaviors (e.g., voice) that can help to identify and understand influences of team phenomena (Waller and Kaplan 2018).

Another aspect is the differentiation between communication (i.e., voice) frequency and quality (Marks et al. 2000). More specifically, in the present review, voice frequency as an outcome is associated with the aggregation of voice occurrences (Kolbe et al. 2012; Raemer et al. 2016; Weiss et al. 2018), whereas voice quality is associated with the assertiveness of the respective voice (Friedman et al. 2015; Pattni et al. 2017; Sydor et al. 2013). Frequency measures demonstrate the actual occurrence of voice, which is, notably, not taken for granted in the context of high-reliability environments (Detert and Edmondson 2011; Barshi and Bienefeld 2018). However, these frequency measures provide less information about the explicit formulation of the voice message, whereas quality measures evaluate how assertively the sender has expressed his or her voice message. By investigating the assertiveness of voice, assertiveness implicitly refers to a counterpart (e.g., an attending anaesthesiologist; Pian-Smith et al. 2009). With that, assertiveness measures of voice address one important factor: the likelihood of being heard by the recipient.

### Recipient of voice

Apart from a few exceptions, our findings indicate that the recipient of voice was not clearly defined in either definitions or measures. More specifically, most studies did not explicitly clarify *who* the potential recipient can be (i.e., higher status vs. peer) or if the recipient is a single person or a group (i.e., dyadic communication vs. team communication).

Previous research considers voice as challenging the status quo (Tangirala and Ramanujam 2012; Wilkinson et al. 2019), which refers to situations in which those who bear responsibility might feel offended (Detert and Burris 2007). In action teams, hierarchical differences are strongly emphasized through authority gradients, such as for example between nurses and physicians in healthcare (Weiss et al. 2017) or between first officers and captains in aviation (Bienefeld and Grote 2014). These authority gradients in action teams are considered to be significant barriers for the expression of voice (Friedman et al. 2015; Klein et al. 2006). Taken together, the concept of voice in action teams might be difficult to delineate from a hierarchical relationship between sender and recipient. Nevertheless, our results showed that more than half of the studies reviewed do not distinguish between peers or higher-status persons as potential voice recipients (Islam et al. 2019; McClean et al. 2018). This is problematic, as research findings that refer to different recipients are difficult to compare. Voicing concerns toward a peer might be easier because hierarchical barriers disappear. This could lead to a higher level of psychological safety of the sender that in turn facilitates the expression of voice (Edmondson 2003).

Another aspect regarding the recipient of voice concerns clarification of the number of persons being addressed, ranging from one single person (Martínez-Córcoles et al. 2018) to the whole group (Islam et al. 2019). The number of recipients might affect not only the sender (Morrison 2011) but also the recipient of voice. From a sender’s perspective, multiple recipients (i.e., a whole group) could increase the barrier against speaking up, particularly if the group is composed of only higher-status persons who are associated with dominance (Islam and Zyphur 2005). On the other hand, if the group is widely composed of peers, perceived peer support might facilitate the expression of voice (Morrow et al. 2016). Moreover, from the recipients’ perspective, voice as “talking to the room”—that is, no explicit recipient is addressed but the room at large (Burtscher et al. 2018a, b)—could lead to a diffusion of responsibility (Kolbe et al. 2014), which in turn could lead to delayed responses. Taken together, the number of recipients might affect the sender’s voice on several levels.

Neither self-report studies nor behavioral observation studies considered the reaction of the voice recipient as a distinct construct. As a notable exception, Schwappach and Richard (2018) mentioned the reaction of the recipient in two items of their scale “Psychological safety for speaking up”, and Kolbe et al. (2012) observed the reaction of the recipient in terms of changes in coordination behavior. However, the recipient’s reaction is important, because for one, the recipient contributes to the outcome of voice (Burris et al. 2017) and for another, the recipient’s reactions could affect the likelihood of subsequent voice (King et al. 2019). Although the recipient’s reaction has been investigated with regard to the sender’s evaluation as an outcome (Burris et al. 2013; Crant et al. 2010; Whiting et al. 2012), there are still hardly any studies available on immediate outcomes of the recipient’s reaction, such as the implementation or rejection of voice. This might be particularly important in the action team context, as the implementation or rejection of voice is likely to have significant consequences, ranging to decisions concerning life-threatening crises (Sydor et al. 2013). Moreover, the recipient’s reaction will probably affect subsequent voice, whether voiced suggestions were endorsed or not (King et al. 2019). In particular, adequate responses in terms of interpersonal fairness, including respectful treatment of the sender (Tangirala and Ramanujam 2009), could more likely lead to subsequent voice. In sum, although adequate responses to voice seems to be crucial for the whole communication process, there is there are barely any studies examining the recipient’s reaction to voice.

### Implications

Our review contributes to the investigation of voice in action teams and has several implications. First, we provide a critical perspective on the treatment of voice in previous action team literature. The research suffers from heterogeneous conceptualizations and measurements, which indicates a need for a more standardized approach to voice research. Studies would benefit from explicitly defining voice variables. Furthermore, stronger consensus among studies, including conceptual approaches (e.g., definitions and the role of the recipient) and methodological approaches (e.g., standardized and validated instruments) would be important to increase the validity and comparability of results of investigations of voice in action teams.

Second, our review offers the opportunity to gain insight into the measurement instruments that have been used to assess voice in action teams. This contributes to an overview of existing methodological approaches and thereby supports the planning of future studies. Moreover, the overview might provide information on the advantages and disadvantages of instruments, indicating that behavioral observation could be more suitable than self-report (Noort et al. 2019) when examining voice in practice.

Third, training and interventions could benefit from the distinction between voice contents, such as promotive (e.g., suggestions and ideas) and prohibitive voice (e.g., preventing hazardous situations; Liang et al. 2012). Depending on the team situation (e.g., emergency situation), prohibitive voice might be more urgent and trainees could be encouraged to express it more assertively than promotive voice. As adequate responses to voice (e.g., leader’s explanation for rejecting voice) would be valuable with regard to promoting subsequent voice (King et al. 2019), it could be beneficial to organizations to train not only the sender’s voice but also the recipient’s response to critical feedback. Taking a step back, research on the reaction to voice itself would be valuable, including the question of what makes a reaction a good reaction. In sum, we would recommend a stronger focus on the recipient of voice. Finally, to investigate effects of the recipient’s response to voice, considering voice as dynamic team process would be useful. In this context, we suggest that voice research would benefit from better integrating emergent states such as team mental models or intra-team trust, which have been shown to play an important role in action teams (Burtscher and Manser 2012; Burtscher et al. 2018a, b). For example, the dynamics of voice and its relationship to team performance may well be contingent on the level of trust among team members.

### Limitations and future research

Our work has several limitations. First, our study focused on voice in action teams and did not involve other team settings. Broadening the range of literature would have led to a more comprehensive conceptual and methodological overview. However, by highlighting the importance of voice in action teams, we concentrated on research in high-reliability environments. Second, we are aware of potential search terms that we did not employ in our literature search. For example, there are similar concepts to voice such as assertiveness or assertive communication that have been associated with error detection and challenging authority in earlier aviation literature (Chute and Wiener 1996; Orasanu et al. 1998). However, we decided to strictly follow the inclusion criteria and explicitly focus on the established concept of voice (Morrison 2011, 2014). Third, teams that work in high-reliability organizations, such as in healthcare, aviation, or military settings, do not necessarily always involve actual action team work. For example, questionnaires that are sent through wards in hospitals might be answered by individuals that do not deal directly with emergency situations. Another aspect leading to the limited generalizability of our results concerns the reviewed studies that were—apart from a few exceptions—mainly conducted in the healthcare domain. Finally, we investigated different types of voice measures but did not rate the quality of methods that were used (Prisma Checklist; Moher et al. 2009). However, the present review did not focus on evaluation of single studies, but instead aimed to provide a bigger picture of how voice has been treated conceptually and methodologically in action team literature.

In addition to the issues mentioned above, future research on voice in action teams might profit from considering the role of non-verbal behavior—both in expressing concerns as well as in receiving voice. In the context of healthcare action teams, non-verbal behaviors such as monitoring other team members (Burtscher et al. 2001) and hand gestures (Härgestam et al. 2016) have been shown to be important aspects of teamwork. Against this background, it would be worthwhile to investigate how specific hand gestures might relate to voice expression and effectiveness.

## Conclusion

This is the first systematic review of the literature on voice in action teams, which also critically considers the treatment of voice in practice. First, our review highlights that concepts and methods are very heterogeneous. For this reason, we would like to emphasize the need for developing a shared understanding of voice in future studies such that a better comparability and integration of research findings will be possible. Second, by showing which instruments have been employed to measure voice, we hope to contribute to an overview—especially for practitioners—that supports future research on voice in action teams. Finally, our review indicates that high-reliability organizations could benefit from conceptualizing voice as a dynamic team process, which includes considering both sender as well as recipient. We hope that this review contributes to a more complete understanding of voice in action teams, particularly regarding the challenges associated with investigating voice in field settings. Ideally, the review should help closing the gap between research and practice and thus contribute to error reduction and improved organizational performance.

## Appendix I Overview: voice definitions

**Table:**

Author, year	Voice definition
Bienefeld and Grote (2012)	“Speaking up in high-risk contexts is defined as an upward voice directed from lower to higher status individuals within and across teams, that challenges the status quo, to avert or mitigate errors” (p. 1)
Bienefeld and Grote (2014)	“Speaking up is defined as un upward voice, directed from subordinates to superiors, that challenges actions or decisions of superiors”^a^ (p. 930)
Pian-Smith et al. (2009)	No definition
Kolbe et al. (2012)	“In accordance with the literature, we define speaking up as explicitly communicating task-relevant observations, requesting clarification, or explicitly challenging or correcting a task-relevant decision or procedure”^a^ (p. 2)
Sydor et al. (2013)	No definition
Weiss et al. (2014)	“By combining Morrison’s (2011) and Edmonson’s (1999) definition of speaking up, we use both terms interchangeably and defined speaking up as explicit communication of suggestions, problems, opinions or doubts that challenge the status quo” (p. 291)
Robb et al. (2015)	No definition
Friedman et al. (2015)	No definition
Raemer et al. (2016)	No definition
Beament and Mercer (2016)	“For the purposes of this article, we define ‘speaking up’ as communicating other team members’ doubts, differing opinions or potential problems about decision or course of action in medical care” (p. 1332)
Pattni et al. (2017)	No definition
Weiss et al. (2017)	“We extended the Co-ACT^c^ speaking up code with five speaking up subcodes: (1) suggestion; (2a) problem, (2b) problem with explicit stop, (3) doubt, (4) opinion. These subcodes are in accordance with recent definitions of voice and reflect the multiple ways with which individuals can speak up”^a,b^ (p. 72)
Weiss et al. (2018)	“The term Speaking up refers to an upward voice, that is, voice from those further down the hierarchy that challenges the actions und opinions from those further up. We will use the terms voice and speaking up interchangeably referring to suggestions, ideas, opinions or concerns from employees that challenge the status quo”^a^ (p. 390)
Oner et al. (2018)	No definition
Farh and Chen (2018)	“Defined as the extent members express suggestions, concerns, or information about a team’s task^1^, member voice is thought to promote team functioning” (p. 97)
Kobayashi et al. (2006)	No definition
Belyanski et al. (2011)	No definition
O’Connor et al. (2013)	“More than just a willingness to question an attending or consultant (senior) physician, speaking up also relates to an individual’s willingness to inform attendings when they are struggling or stressed or when they have made an error” (p. 426)
Schwappach and Gehring (2014)	No definition
Martinez et al. (2015)	“We defined ‘speaking up’ as stating concerns (e. g, filing a report, sharing concerns with a supervisor or speaking directly with the individual(s) involved) rather than saying nothing” (p. 1)
Martinez et al. (2017)	“Open communication regarding concerns, also known as ‘speaking up’, is vital to keeping patients safe and preventing errors”^d,e,f,g^ (p. 869)
Schwappach and Richard (2018)	“Speaking up is defined as assertive communication of patient safety concerns through information, questions or opinions where immediate action is needed to avoid patient harm”^h,i^ (p. 827)
Islam et al. (2019)	“Employee voice is the employees’ response in terms of suggestions, opinions, concerns and ideas about work-related issues with intentions to improve the working environment and overall organizational processes. It denotes an individual’s tendency vigorously talk about change and productive ideas. More specifically, voice is defined as a “behavior that emphasizes expression of constructive challenge with the intent to improve rather than merely criticize”^j^. Voice behavior is socially based as it promotes change by facilitating constructive change intentions. However, it is to share that challenging the status quo and raising voice for change create social risks”^k^(p. 4)
Alingh et al. (2019)	“Healthcare professionals who question clinical practices that may compromise patient safety and who raise ‘*concerns* […] *upon recognising or becoming aware of the risky or deficient actions of others within healthcare teams’* can prevent the occurrence of adverse events, improve team performance and facilitate a learning environment”^b,d,e^ (p. 39)
McClean et al. (2018)	“Employee voice is a change-oriented behavior involving the discretionary provision of improvement-oriented information intended to better one’s group or organization”^l^ (p. 1869)
Martínez-Córcoles et al. (2018)	“Critical upward communication is an essential feature of effective team performance in extreme contexts. It refers to explicitly communicating task-relevant observations, requesting clarification, and/or explicitly questioning a task-relevant decision or a procedure”^a,d^ (p. 6)

^a^Morrison (2011); ^b^(2014); ^c^Kolbe et al. (2013); ^d^(2012); ^e^Okuyama et al. (2014); ^f^Srivastava (2013); ^g^Leonard et al. (2004); ^h^Schwappach and Gehring 2014; ^i^Lyndon et al. (2012); ^j^Van Dyne and LePine (1998); ^k^LePine and Van Dyne (2001); ^l^Detert and Burris (2007).

## Footnotes

1 For simplicity, we use "voice" as an umbrella term.

2 We found 26 articles, each containing one study. McClean et al. (2018) represents an exception, as it includes two studies. In our review, we refer to McClean et al.'s study 1, as it is the only one that is set in an action team context (and therefore fulfills our selection criteria).
